# 

**Published:** 1901-05

**Authors:** 


### The Brazos Valley Medical Association.

This association will hold its eleventh semi-annual meeting at Calvert, Texas, on the 14th and 15th of May, 1901. Election of officers will take place at this meeting, and we hope to have every member present. Let us make it, if possible, the most interesting meeting ever held by the association. Mutual co-operation of all members is imperative. To those who have written articles we would say, if you find it impossible to attend, be sure to send your papers to the secretary by the 10th inst., that they may be read before the association.

J. P. OLIVER, President.

W. B. BRIGGS, Secretary.

A lengthy and most attractive program has been prepared. We regret that press of matter prevents our giving it in full. A large attendance and a royal good time are anticipated.

### East Texas Medico-Chirurgical Society.

This flourishing society will hold its third semi-annual meeting at Palestine, Texas, Thursday and Friday, May 30-31, inst. We have received the program; it is very attractive and complete, and we would give it in full were not our limited space so completely taken up with State Association matters and other papers. The writer expects to be present both at this meeting and that of the Brazos Valley society, May 12, and anticipates a good time, as among the members of both are hosts of warm personal friends and staunch supporters of the "Red-Back," their official organ.

The officers of the East Texas Medico-Chirurgical Society are: Dr. Sam R. Burroughs, Buffalo, president; Dr. J. H. Evans, Palestine, first vice-president; Dr. W. C. Lipscomb, Crockett, second vice-president; Dr. John Colley, Palestine, treasurer; Dr. E. E. Guinn, Rusk, secretary.

### American Congress of Tuberculosis.

It is announced that the second annual meeting of the American Congress of Tuberculosis will be held at the Grand Central Palace, in the city of New York, on the 15th and 16th days of May, 1901, in joint session with the Medico-Legal Society of New York. That a dinner will be given to the members and guests. It is proposed to open a museum of pathology, bacteriology and public health, with an exposition of electrical and other instruments, with the use of the power furnished at the building, which it is intended to be made most complete, educating and attractive, of all appliances used in any way in arrest or treatment of the disease.

The leading manufacturers have enlisted already, many of them, and the display will be on an extensive scale. The objects of the congress will be to exchange the information and experience gained throughout the world as to forces and methods most available for the extermination of consumption, which at the present moment is a disease the most destructive of human life of any that now afflicts humanity.

The medical profession of all countries will be invited to contribute papers to be read before this congress in their behalf by a committee selected for that purpose in case of the inability of the author to attend, and to enable those who could not hope or expect to be present, to participate in the work and usefulness of the body. As the questions to be discussed involve remedial legislation, legislators, lawyers, judges, and all publicists, who take an interest in the subject, are also invited, both to enroll and contribute papers.

The papers should be forwarded to the secretary on or before the 15th day of April, next, and the title of the papers forthwith, to facilitate classification, as the time is short. The enrolling fee will be $3, entitling the member to the bulletin of the transactions free.

The complete list of officers and committees will be announced as early as possible. The preliminary announcement is now made to obtain the names of those who will co-operate in the congress, and an early classification of the subjects and titles.

